# Characterization of FosA13, a novel fosfomycin glutathione transferase identified in a *Morganella morganii* isolate from poultry

**DOI:** 10.3389/fcimb.2025.1534084

**Published:** 2025-03-11

**Authors:** Runzhi Zhang, Yan Yu, Lulu Huang, Susu Chen, Ruxi Hu, Xiuxiu Wang, Dawei Huang, Chunhan Song, Junwan Lu, Qiyu Bao, Yunliang Hu, Pengfei Jiang, Wei Pan

**Affiliations:** ^1^ Institute of Molecular Virology and Immunology, Department of Microbiology and Immunology, School of Basic Medical Sciences, Wenzhou Medical University, Wenzhou, China; ^2^ Institute of Biomedical Informatics/School of Laboratory Medicine and Life Sciences, Wenzhou Medical University, Wenzhou, China; ^3^ The Second Affiliated Hospital and Yuying Children’s Hospital, Wenzhou Medical University, Wenzhou, China; ^4^ Department of Laboratory Sciences, Pingyang Hospital of Wenzhou Medical University, Pingyang, China; ^5^ Department of Laboratory Sciences, The People’s Hospital of Yuhuan, Yuhuan, China; ^6^ Medical Molecular Biology Laboratory, School of Medicine, Jinhua University of Vocational Technology, Jinhua, China

**Keywords:** *Morganella morganii*, resistance gene, *fosA13*, fosfomycin glutathione transferase, kinetic parameter

## Abstract

**Background:**

*M. morganii* is a species of the genus *Morganella* in the family *Enterobacteriaceae*. This species primarily causes infections of postoperative wounds and the urinary tract. Some isolates of *M. morganii* exhibit resistance to multiple antibiotics due to multidrug resistance traits, complicating clinical treatment; thus, there is a growing need to elucidate the resistance mechanisms of this pathogen.

**Methods:**

A total of 658 bacterial strains were isolated from anal fecal swabs from poultry and livestock and from the surrounding environment in Wenzhou, China, via plate streaking. The full genome sequences of the bacteria were obtained via next-generation sequencing platforms. The standard agar dilution method was employed to determine the minimum inhibitory concentrations (MICs) of various antimicrobial agents. The resistance gene (*fosA13*) of the isolate was identified using the Comprehensive Antibiotic Resistance Database (CARD) and confirmed via molecular cloning. The FosA13 protein encoded by the novel resistance gene *fosA13* was expressed with the vector pCold I, and its enzyme kinetics parameters were characterized. The genetic background and evolutionary process of the sequence of this novel resistance gene were analyzed by means of bioinformatics methods.

**Results:**

In this study, we identified a new chromosomally encoded fosfomycin resistance gene, designated *fosA13*, from the *M. morganii* isolate DW0548, which was isolated from poultry on a farm in Wenzhou, China. Compared with the control strain (pUCP19/DH5α), the recombinant strain carrying *fosA13* (pUCP19-*fosA13*/DH5α) presented a fourfold increase in the MIC value for fosfomycin. The enzyme kinetics data of FosA13 revealed effective inactivation of fosfomycin, with a *k*_cat_ */K*_m_ of (1.50 ± 0.02)×10^4^ M^-1^·s^-1^. Among functionally characterized resistance proteins, FosA13 presented the highest amino acid (aa) homology (55.6%) with FosA. FosA13 also contained essential functional residues of FosA proteins. The isolate *M. morganii* DW0548 presented high MIC values (≥ 8 μg/mL) for 5 classes of antimicrobials, namely, aminoglycosides, β-lactams, quinolones, tetracycline, and chloramphenicol, but only two functionally characteristic antimicrobial resistance genes (ARGs) have been identified in the complete genome: a β-lactam resistance gene (*bla*_DHA-16_) and a phenol resistance gene (*catII*). These findings indicate that in addition to the novel resistance gene identified in this work, other uncharacterized resistance mechanisms might exist in *M. morganii* DW0548.

**Conclusion:**

A novel chromosomal fosfomycin resistance gene, *fosA13*, was identified in an animal *M. morganii* isolate, and its enzymatic parameters were characterized. This protein shares the highest aa identity of 55.6% with the functionally characterized protein FosA and has all the essential functional residues of FosA proteins. Exploring more antimicrobial resistance mechanisms of this pathogen would help clinicians choose effective drugs to treat infectious diseases in animal husbandry and clinical practice and facilitate the development of methods to prevent the spread of resistance between bacteria of different species.

## Introduction

In 1969, a natural antibiotic named fosfomycin was first discovered in the fermentation broth of *Streptomyces* (Hendlin et al., 1969). Although some species can produce fosfomycin, its concentrations are generally low. Fosfomycin exhibits bactericidal properties against various bacteria, including both Gram-negative and Gram-positive bacteria such as staphylococci (Raz, 2012). Fosfomycin was a decommissioned antibiotic, however, given the increasing prevalence of multidrug-resistant uropathogens, the limited treatment options available, and the lack of new antibiotics, older antibiotics need to be reevaluated. Owing to its unique bactericidal mechanism and physicochemical properties, fosfomycin has the advantages of no cross-resistance, strong antibacterial activity, a wide tissue distribution, and synergistic bactericidal effects when used in combination with other drugs, and was defined by the World Health Organization (WHO) as a “vital” antibiotic (Collignon et al., 2016), which has attracted the interest of many clinicians. With an increase in the frequency of clinical fosfomycin use, resistance to fosfomycin has also increased in some bacteria, such as *Acinetobacter*, *Vibrio fischeri*, *Chlamydia trachomatis*, and so on (Silver, 2017).

The earliest case of fosfomycin resistance dates back to 1977. Since then, there have been epidemics of drug-resistant strains in all countries worldwide (Aghamali et al., 2019). Although the mechanism of action and the structure of fosfomycin are unique, which made the cross-resistance uncommon (Falagas et al., 2020), with the increased use of fosfomycin, bacterial resistance to it has also increased rapidly. Data show that the use of fosfomycin in the treatment of urinary tract infections caused by *Escherichia coli*, as well as in the treatment of some uropathogen infections, leads to an increase in fosfomycin resistance (Jiang et al., 2015).

Several categories of fosfomycin drug resistance mechanisms have been characterized (Karageorgopoulos et al., 2012). These mechanisms involve reducing drug absorption, altering drug binding targets, and inactivating fosfomycin. Resistance to fosfomycin is typically associated with the inactivation of fosfomycin by modifying enzymes (such as *fosA*, *fosB*, *fosC*, and *fosX*) (Yang et al., 2019; Findlay et al., 2023), and kinases (*fomA* and *fomB)* (Kobayashi et al., 2000). FosA is a dimeric glutathione S-transferase (GST) that catalyzes the binding of glutathione to fosfomycin with the help of Mn^2+^ and K^+^ ions, inactivating fosfomycin, and it can be encoded on either a plasmid or a chromosome (Suárez and Mendoza, 1991). FosA was first found to be present in the plasmid Tn*2921* transposon of *Serratia marcescens*, and it is predominantly present in Enterobacteriaceae, *Pseudomonas* spp (Biggel et al., 2021). The FosA proteins are a number of metalloenzymes able to disrupt the epoxide ring of fosfomycin. It depends on manganese (II) and potassium as cofactors, and glutathione (GSH) as a nucleophilic molecule (Mattioni Marchetti et al., 2023). The *fosA* genes are commonly distributed in *Providencia stuartii*, *Providencia rettgeri*, *Klebsiella pneumoniae*, *Klebsiella oxytoca*, *Serratia marcescens*, *Enterobacter aerogenes* and *Enterobacter cloacae*, however, they are rarely reported in *Citrobacter freundii*, *Proteus mirabilis* and *Acinetobacter baumannii* (Zurfluh et al., 2020). FosB is an L-cysteine thioltransferase that inactivates fosfomycin via the nucleophilic addition of thiols to fosfomycin with the help of Mg^2+^ (Michalopoulos et al., 2011). FosC is a protease inactivating fosfomycin by adding a phosphate group to it by using ATP as a substrate (García et al., 1995). FosX is a Mn^2+^-dependent epoxide hydrolase that inactivates fosfomycin by adding a hydroxyl group to the fosfomycin C1 position and opening its epoxide ring, using water as a substrate (Rigsby et al., 2005). FomA and FomB are kinases involved in the degradation of fosfomycin, and the role of these kinases may be to protect fosfomycin producers from fosfomycin (Kobayashi et al., 2000). Chromosomal mutations can affect the transport function of the cell membrane, leading to reduced intracellular levels of fosfomycin. Two transport systems for the uptake of fosfomycin into cells, which involve glycerol-3-phosphate transporter protein (GlpT) and hexose phosphate transporter protein (UhpT), are present in *E. coli* (Takahata et al., 2010). Altering the drug’s target of action is another mechanism of fosfomycin resistance. Among Gram-positive bacteria, the affinity between the MurA protein and fosfomycin in *S. aureus* is reduced by mutations in the *murA* gene (Xin et al., 2022); however, elevated expression levels of this gene can lead to bacterial resistance to fosfomycin (Raina et al., 2021).

*M. morganii*, a pathogen that was first isolated from pediatric fecal cultures by Morgan et al. in 1906 (Morgan and de, 1906), is a parthenogenetic anaerobic rod-shaped Gram-negative enteric bacterium that produces virulence factors such as hemolysin and causes urinary tract wound infections, and the risk of infection by *M. morganii* has been highlighted in many epidemiological data. Clinical disease caused by multidrug-resistant (MDR) or even extensively drug-resistant (XDR) *M. morganii* often results in treatment failure. Studies have shown that the development of intrinsic and acquired multidrug resistance is of concern as the prevalence of *M. morganii* infections increases, necessitating the global identification of *M. morganii* as a major pathogen (Liu et al., 2016a; Bandy, 2020a; Li et al., 2023). Rising rates of *M. morganii* infections are a reminder not only of the need for increased precautions in public areas but also of the need to include this microorganism in the differential diagnosis list in the clinical setting (Bandy, 2020).

In this work, we report a novel chromosomally encoded fosfomycin gene, *fosA13*, in the isolate DW0548, which was isolated from a farm animal. DW0548 was subjected to whole-genome sequencing for genome-wide characterization. To determine the function of the *fosA13* gene, molecular cloning was performed. The protein encoded by *fosA13* was expressed, and its enzyme kinetics were also analyzed.

## Materials and methods

### Origin of bacterial strains and identification of species

To analyze the drug resistance status of bacteria isolated from animals and the environment of the animal farms, 658 bacterial strains were obtained from the poultry and livestock anal fecal swabs, and sewage and soil of the animal farms in Wenzhou, China. An *fosA*-like gene, designated *fosA13*, was found in an isolate from poultry named DW0548. Bacterial species identification was performed via 16S rRNA gene homology and genome-wide average nucleotide identity (ANI) analyses. **Table 1** lists the plasmids and strains used in this study.

**Table 1:** Bacteria and plasmids used in this work.

Bacteria and plasmids	Description	Source
DW0548	The wild type of *Morganella morgani* DW0548	Chicken
DH5α	*E. coli* DH5α as a host cell for cloning of the *fosA13* gene	Our laboratory collection
pUC19	Cloning vector for the PCR product of *fosA13* with its upstream promoter region, AMP^r^	Our laboratory collection
ATCC25922	*E. coli* ATCC 25922 as quality control for MIC testing	Our laboratory collection
BL21	*E. coli* BL21 as a host cell for the expression of FosA13	Our laboratory collection
pColdI	Expression vector for the PCR product of the ORF of the *fosA13* gene, AMP^r^	Our laboratory collection
pUCP19-*fosA13*/DH5α	The DH5α cell carrying recombinant plasmid pUCP19-*fosA13*	This research
pCold I-*fosA13*/BL21	The BL21 cell carrying the recombinant plasmid pCold I-*fosA13*	This research

r , resistance; AMP, ampicillin; ORF, open reading frame.

### Antibiotic susceptibility testing of the bacteria

The MICs of the antimicrobials were determined via the agar dilution method according to Clinical Laboratory Standards Institute (CLSI) guidelines (CLSI, 2024). Medium with glucose-6-phosphate (G6P) at a constant concentration of 25 μg/mL was used when the MIC of fosfomycin was tested. *E. coli* ATCC 25922 was used as a quality control. **Table 2** shows the MIC data for the 25 antibiotics from the six antibacterial categories.

**Table 2:** The MIC results of 25 antimicrobials for 5 strains (μg/mL).

Drug class	Antimicrobials	ATCC25922	DH5α	pUCP19/DH5α	pUCP19- *fosA13*/DH5α	*M. morganii* DW0548
Aminoglycosides	Gentamicin	0.5	8	0.5	/	1
	Tobramycin	0.5	0.5	0.5	/	0.5
	Streptomycin	4	2	2	/	8
	Kanamycin	8	8	8	/	8
	Spectionmycin	8	64	8	/	32
	Paromomycin	4	4	8	/	8
	Neomycin	1	16	8	/	2
	Sisomicin	≤1	≤1	≤1	/	≤1
	Amikacin	≤2	≤2	≤2	/	≤2
	Netilmicin	≤0.125	≤0.125	≤0.125	/	≤0.125
	Ribostamycin	4	≤2	≤2	/	4
β-Lactams	Cefazolin	2	1	16	/	512
	Cefothiophene	32	≤4	64	/	>1024
	Cefoxitin	4	4	4	/	16
	Cefuroxime	16	16	16	/	16
	Ceftazidime	0.5	1	1	/	0.5
	Cefotaxime	0.5	≤0.06	0.125	/	0.125
	Ceftriaxone	0.125	8	0.125	/	0.0625
	Cefepime	0.03	0.015	0.0625	/	0.03
Quinolones	Levofloxacin	≤0.05	≤0.05	≤0.05	/	≤0.05
	Nalidixic acid	64	32	64	/	64
Tetracycline	Tetracycline	1	2	1	/	32
Phosphonic acid derivative	Fosfomycin	0.5	0.5	0.5	2	2
Amphenicols	Chloramphenicol	16	16	16	/	64
	Florfenicol	4	8	4	/	4

### Whole-genome sequencing and functional analysis

The bacteria were cultured in liquid LB medium and incubated at 37°C for 16 h. Total DNA was extracted from the bacteria with the Generay Genomic DNA Small Volume Preparation Kit (Shanghai Generay Biotech Co., Ltd., Shanghai, China). Whole-genome sequencing was completed on the Illumina NovaSeq and PacBio RS II platforms at Shanghai Personal Biotechnology Co., Ltd. (Shanghai, China). The Illumina short reads and the PacBio long reads were assembled using MEGAHIT v1.2.9 (Li et al., 2016) and Trycycler v0.5.1 (Wick et al., 2021), respectively. Using Pilon v1.24, the quality of the genome sequence from PacBio sequencing was corrected by mapping the Illumina short reads onto the PacBio read assembly (Walker et al., 2014). The open reading frames (ORFs) were predicted with Prokka v1.14.6 (Seemann, 2014). The functions of the predicted proteins were annotated by searching the ORFs against the NCBI nonredundant protein database with DIAMOND v2.0.11 (Buchfink et al., 2021). The promoter region of a gene was predicted using the BPROM tool (http://www.softberry.com/berry.phtml?topic=bprom&group=programs&subgroup=gfindb). Annotation of drug resistance genes was conducted by using Resistance Gene Identifier v5.2.0 (RGI) and the Comprehensive Antibiotic Resistance Database (CARD) database (https://github.com/arpcard/rgi) (McArthur et al., 2013). Homology analysis of the 16S rRNA gene from the target genome was performed by comparison with the 16S ribosomal RNA sequence database in NCBI (McArthur et al., 2013). The ANI was computed using FastANI v1.33 (Jain et al., 2018). Calculation of digital DNA−DNA hybridization (dDDH) values was performed on the basis of the Type strain Genome Server (TYGS) online database (Type Strain Genome Server) (Lian et al., 2021). Multiple sequence alignment of *fosA13*, *fosA*, *fosA2*, and other related genes was performed with MAFFT v7.487 (Katoh and Standley, 2013). A neighbor-joining (N-J) phylogenetic tree including FosA13 and other functionally characterized fosfomycin resistance enzymes was constructed with MEGA11 (Kumar et al., 2018). The phylogenetic tree of FosA13 with other Fos proteins was visualized with the online website iTol (iTOL: Interactive Tree Of Life) (Letunic and Bork, 2021).

### Molecular cloning of the identified resistance gene

Referring to a previous publication (Zhao et al., 2023), primers to clone the predicted resistance gene with its promoter region and the primers to clone the open reading frame (ORF) of the gene were designed. The PCR products were amplified and then inserted into the vectors pUCP19 and pCold I, respectively, via the DNA ligation kit Ver.2.1 (Takara Bio, Inc., Dalian, China). The recombinant plasmids pUCP19-*fosA13* and pCold I-*fosA13* were transformed into *E. coli* DH5α and *E. coli* BL21, respectively. The inserted sequences in the recombinants were verified by Sanger sequencing. **Table 3** shows the primer sequences and details.

**Table 3:** Primers for cloning the *fosA13* gene.

Primer^a^	Sequence(5´-3´)^b^	Restriction endonuclease	Vector	Annealing temperature (°C)	Amplicon size (bp)
pro-*fosA13*-F	TCAGTTCCATAACAGTGAGAAAGCC	/	pUCP19	58	879
pro-*fosA13*-R	TAGCAGTGTCTCCGTAAGATAAGGG	/	pUCP19	59	879
orf-*fosA13*-F	CGGGGTACCGACGACGACGACAAGATGTTAACAGGAATGAATCATCTG	KpnI	pColdI	54	451
orf-*fosA13*-R	CCCAAGCTTGATAAGGGTTTATCCCATCTTACAC	HindIII	pColdI	55	451

a Primers with “pro” were used to clone the *fosA13* gene with its promoter region, and primers with “orf” were used to clone the ORF of the *fosA13* gene.

b The underline sequences represent the restriction sites and their protective bases.

### Expression and purification of the recombinant FosA13 protein

The methods used to express and purify the recombinant FosA13 protein were mainly based on a previous publication (Gao et al., 2022). In brief, the recombinant strain (pCold I-*fosA13*/BL21) was cultured in LB broth. When the OD value of the culture reached between 0.45 and 0.55, the expression of the protein was induced by 1 mM isopropyl-β-dithiogalactopyranoside (IPTG). Bacteria were harvested by centrifugation (4,000 × g, 10 min) at 4°C, resuspended in 5 mL of nondenaturing lysis buffer, and fragmented by ultrasonication for 10 min at 4°C. After centrifugation, the supernatant containing the recombinant protein was collected. The recombinant protein was purified with BeyoGold His-tag purification resin using a nondenaturing elution solution from a His-tag protein purification kit (Beyotime, Shanghai, China). The His-tag of the recombinant protein was removed by enterokinase (EK enzyme). The purity of the protein was determined via SDS−PAGE, and the protein concentration was subsequently determined using a BCA protein concentration assay kit (Beyotime, Shanghai, China).

### Enzyme kinetics studies of FosA

The kinetic parameters of purified FosA13 with fosfomycin were analyzed via high-performance liquid chromatography (HPLC) using a Thermo Scientific AcceLA HPLC system (Thermo Fisher Scientific, Inc., China) with a 100 μL final reaction volume at 37°C. The 100 μL reaction system consisted of 10 mM GSH, 250 µm MnCl_2_, 100 mM KCl, and 100 mM Tris-HCl, and gradient concentrations of fosfomycin (0, 25, 50, 100, 200, 400, and 800 µM) were added in a volume of 10 μL. After the reaction system was preheated for 20 minutes, the purified FosA13 protein was added. The reaction was conducted for 5 minutes, and the assay was carried out on the analysis system. The mobile phases were a mixture containing K_2_HPO_4_ (200 mM), KH_2_PO_4_ (200 mM), acetonitrile and methanol at a percentage volume ratio of 1.68: 78.32: 10: 10 (Arca et al., 1990; Bernat et al., 1997, Bernat et al., 1999). The steady-state kinetic parameters *k*_cat_ and *K*_m_ were nonlinearly regressed against the initial reaction rate via the Michaelis−Menten equation in Prism (version 8.0.2) software (GraphPad software, CA, United States). The value is the average of three independent measures.

### Nucleotide sequence accession numbers

The GenBank accession numbers for the novel fosfomycin *fosA13* gene, the plasmid pMMDW0548 and the chromosome sequences of DW0548 are PQ600006, CP173708 and CP173707, respectively.

## Results and discussion

### Discovery of a new drug resistance gene

To clarify the mechanisms of antimicrobial resistance in bacteria isolated from animals and the environment, we sequenced 658 bacterial isolates isolated from poultry and livestock anal fecal swabs and environmental samples from animal farms in Wenzhou, China. Annotation of genomic data revealed resistance genes against different antibiotics. Notably, among the predicted genes were numerous putative fosfomycin resistance genes, including but not limited to homologs of *fosA, fosB, fosC*, and *fosX*. These genes shared less than 80.0% aa sequence identity with functionally characterized fosfomycin resistance genes. Some of these genes, including the *fosA2, fosA5*, *fosB*, *fosLC2* and *fosL1* homologs, were randomly selected and cloned, and their resistance functions were determined. Finally, a gene homologous to *fosA2* (designated *fosA13* in this work) that conferred resistance to fosfomycin was identified, and it was encoded in the chromosome of an isolate named DW0548.

### Cloning and phenotyping of the novel resistance gene

To confirm the resistance function of this gene, the ORF with its promoter region was cloned into the vector pUCP19, and the recombinant plasmid containing *fosA13* was transformed into *E. coli* DH5α cells. The transformant strain carrying *fosA13* (pUCP19-*fosA13*/*E. coli* DH5α) showed a 4-fold increase in the MIC value to fosfomycin (2 μg/mL) compared with that of the control strain (pUCP19/DH5α, 0.5 μg/mL) (**Table 2**). The MICs of the previously cloned fosfomycin resistance genes varied. Compared with the recipients, the cloned *fosY* (Chen et al., 2022) and *fosA6* (Guo et al., 2016a) genes increased the MIC levels by 16- and 32-fold, respectively, whereas *fosI* (Pelegrino et al., 2016) and *fosA7* (Rehman et al., 2017) increased the MIC levels by 128- and >256-fold, respectively (**Supplementary Table S1**).

### Classification and genome characterization of the isolate DW0548

16S rRNA gene homology analysis revealed that the 16S rRNA gene of the isolate DW0548 shared the highest similarity (96.0% coverage and 99.0% identity) with that of *M. morganii* M11 (NR_028938.1). In addition, ANI analysis of all 759 *Morganella* genomes in the NCBI database revealed that 78 of these genomes had ≥ 95.0% ANI (the threshold value to define a bacterial species) with the isolate DW0548 genome. Seventy-three of these genomes were from the *M. morganii* genomes. The result of the digital DNA–DNA hybridization (dDDH) analysis revealed that the isolate DW0548 presented the highest dDDH value (75.7%) with *M. morganii* NBRC 3848, which was greater than the threshold (70.0%) for classifying a bacterial species. Therefore, on the basis of the results above, the isolate DW0548 was ultimately included in the species *M. morganii* and was thus designated *M. morganii* DW0548.

The whole genome of *M. morganii* DW0548 consists of a chromosome and a plasmid named pMM548. The chromosome size was approximately 4.31 Mb, and the average GC content was 50.35%, with 4,380 coding sequences (CDSs). The plasmid was 96,349 bp in length with the average GC content of 50.45%, and it encoded 109 CDSs (**Table 4**).

**Table 4:** General features of the *fosA13* genome.

	Chromosome	pMMDW0548
Size (bp)	4,313,176	96,349
GC content (%)	50.35	50.45
Predicted coding sequences (CDSs)	4,380	109
Known proteins	3,151	38
Hypothetical proteins	1,229	71
Protein coding (%)	96.31	99.08
Average ORF length (bp)	868.4	820.7
Average protein length (aa)	288.6	262.3
tRNAs	81	0
rRNA operons	(16S-23S-5S) × 6 (16S-23S-5S-5S) × 1	0

### The resistance profile of *M. morganii* DW0548

The result of the susceptibility test demonstrated that *M. morganii* DW0548 had high MICs (≥ 8 μg/mL) for 11 of the 25 antimicrobials tested, which included members of 5 classes of antimicrobials, with 4 aminoglycosides (streptomycin, kanamycin, spectinomycin and paromomycin), 4 β-lactams (cefazolin, cefothiophene, cefoxitin and cefuroxime), 1 quinolone (naphridixic acid), tetracycline, and chloramphenicol (**Table 2**).

In examining the relationship between the drug resistance phenotype and genotype, we found that even though the bacterium presented high MICs for antimicrobials from the 5 classes tested, only two genes (a β-lactam resistance gene, *bla*_DHA-16,_ and a phenicol resistance gene, *catII*), which shared ≥ 80% aa similarity with functionally characterized antimicrobial resistance genes (ARGs), were identified. No aminoglycoside, tetracycline or quinolone resistance genes were found in the whole-genome sequence (**Supplementary Table S2**). Similar to the isolate DW0548, other *M. morganii* strains have been reported to be resistant to β-lactams, aminoglycosides, tetracyclines, fluoroquinolones, fosfomycin, and other types of antibiotics (Liu et al., 2016).

### Homology analysis of FosA13 with the other FosA proteins

**Figure 1:**
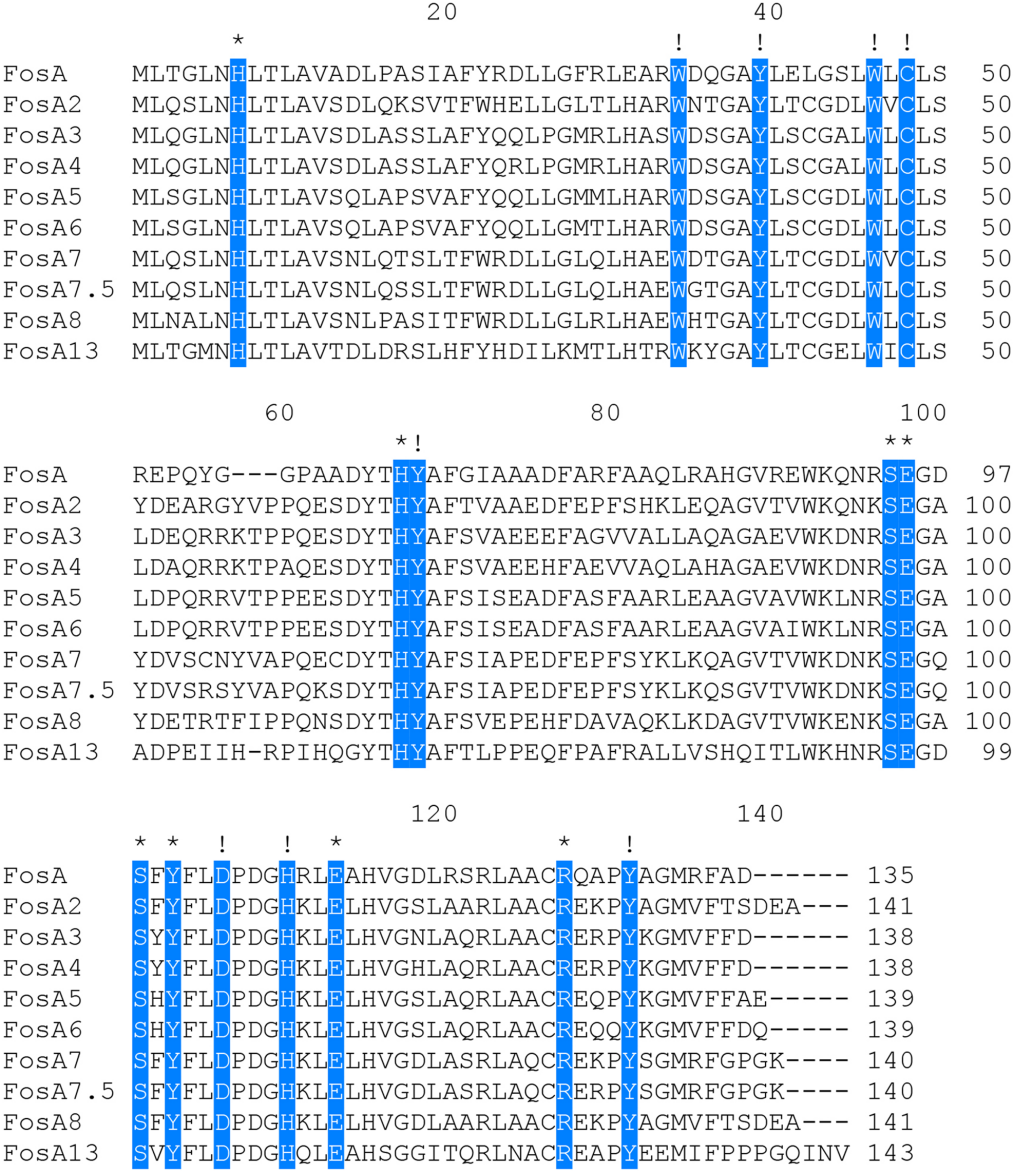
Multiple alignment of the deduced amino acid sequences of FosA13 and its close relatives. The 16 amino acids shaded in blue are function and structure essential residues of FosA proteins, of which those indicated by asterisks are residues that act as ligands for Mn^2+^, K^+^ and fosfomycin and those indicated by exclamation points are residues located in the putative fosfomycin/GSH-binding channel. Spaces are indicated by hyphens. The numbers on the right represent the corresponding sequence lengths.

**Figure 2:**
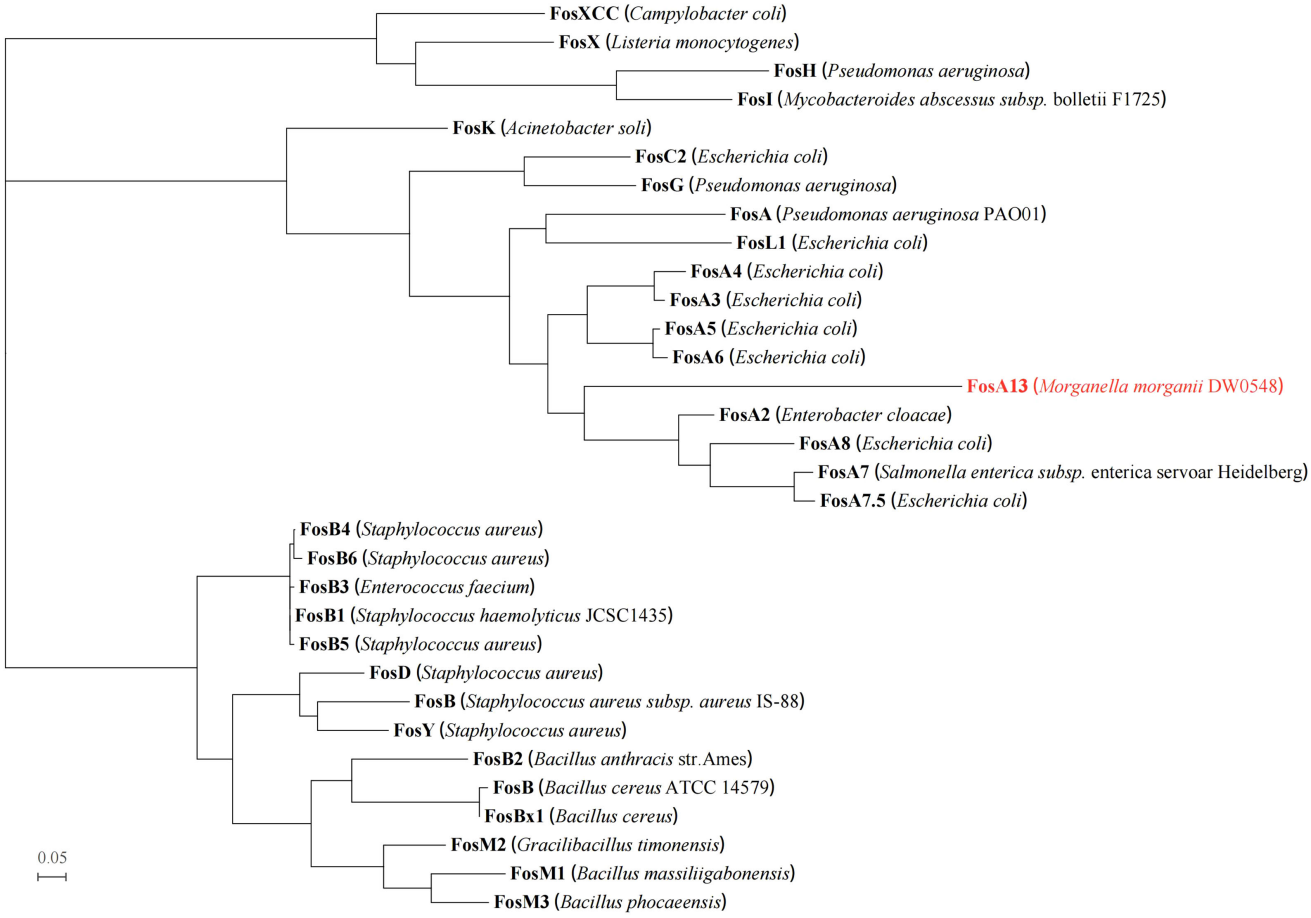
Phylogenetic tree showing the relationship of FosA13 with other functionally characterized proteins (with identities between 20.0% and 60.0%). FosA13 is highlighted in red. The other proteins include FosA (AAG04518.1), FosA2 (ACC85616.1), FosA3 (AEG78825.1), FosA4 (BAP18892.1), FosA5 (AJE60855.1), FosA6 (AMQ12811.1), FosA7 (KKE03230.1), FosA7.5 (ANQ03635.1), FosA8 (QEI22965.1), FosB (EHS19134.1, *S. aureus*), FosB (AAP08996.1, (*B*) *cereus*), FosB1 (BAE05988.1), FosB2 (AAP27834.1), FosB3 (ADX95999.1), FosB4 (ALM24139.1), FosB5 (ALN12426.1), FosB6 (ALM24145.1), FosBx1 (QLF01382.1), FosC2 (BAJ10053.1), FosD (BAG12271.1), FosG (RTB44598.1), FosH (ADF48907.1), FosI (AFJ38137.1), FosK (BAO79518.1), FosL1 (QHR93773.1), FosM1 (DAC85639.1), FosM2 (DAC85640.1), FosM3 (DAC85641.1), FosX (CWV56762.1), FosXCC (AIF29598.1), and FosY (QTE33800.1).

A comparison of FosA13 with these functionally characterized proteins in the CARD revealed that it had the greatest aa sequence similarities with FosA (55.6%), followed by FosA2 (55.2%), FosA3 (55.2%), FosA4 (55.2%), FosA5 (55.2%), FosA6 (55.2%), FosA7 (52.9%), FosA7.5 (52.9%), and FosA8 (52.9%). Sixteen function and structure essential residues of FosA (Beharry and Palzkill, 2005a) are conserved in FosA13. Half of them act as ligands for Mn^2+^ (His7, His64 and Glu110) and K^+^ (Ser94, Ser98 and Glu95) and ligands (Arg119 and Tyr100, within the hydrogen-bonding site of fosfomycin) involved in FosA binding to fosfomycin, and the other half of the residues (W34, Y39, W46, C48, Y65, D103, H107, Y128) were located in the putative fosfomycin/GSH binding channel. Although FosA13 shares only about 50–60% identity with these functionally characterized FosA proteins, the active site residues essential for FosA function remain unchanged (**Figure 1**). Further analysis of the evolutionary relationship between FosA13 and different glutathione transferases revealed that FosA13 was most similar to FosA2 and formed a new branch (**Figure 2**).

### Kinetic parameters of FosA13

The *fosA13* gene is 432 bp in length and encodes a 143 aa protein (FosA13). The predicted molecular weight of the mature glutathione-S-transferase is 16.48 kDa, with a pI of 6.08. The purified FosA13 has the ability to hydrolyze fosfomycin, with a *K*_m_ of 0.427 ± 0.007 µM, *k*_cat_ of 6.43 ± 0.04 s^-1^ and *k*_cat_ */K*_m_ of (1.50 ± 0.02) × 10^4^ M^-1.^ s^-1^. In terms of enzyme kinetics, compared with the other two FosA proteins, FosA13 showed 6-fold lower hydrolytic activity against fosfomycin than that of FosA (Beharry and Palzkill, 2005b) (*k*_cat_ */K*_m_ of 1.5 × 10^4^ M^-1).^ s^-1^ vs. 9.0 × 10^4^ M^-1.^ s^-1^) and approximately 5-fold lower than that of FosA6 (Guo et al., 2016) (*k*_cat_ */K*_m_ of 1.5 × 10^4^ M^-1.^ s^-1^ vs. 0.3 × 10^4^ M^-1.^ s^-1^).

### Distribution and context of the *fosA13*-like genes

**Figure 3:**
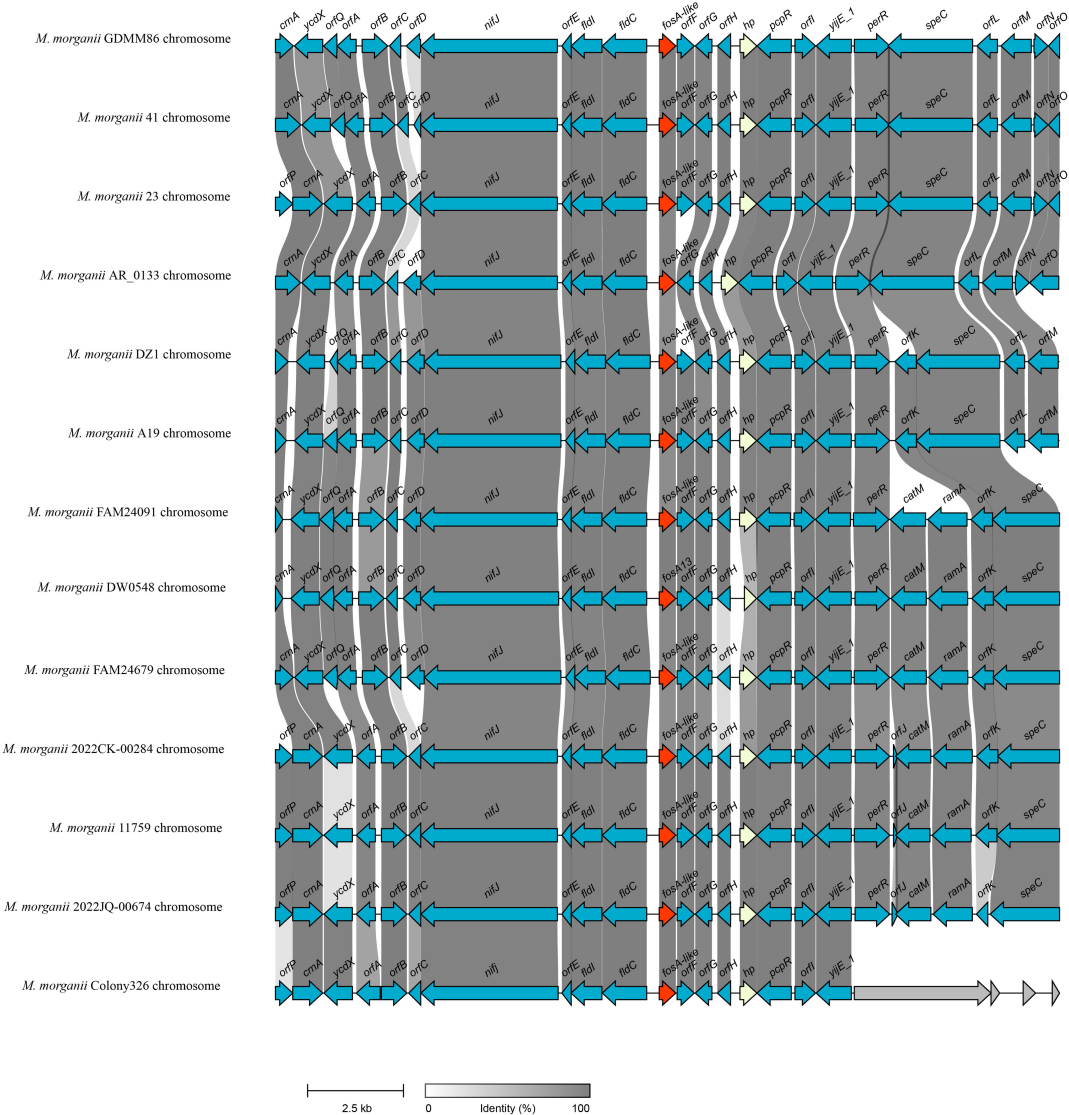
Genetic environment of *fosA13* and *fosA13*-homologous genes. The *fosA* genes are in red. *orfA*, DUF523 domain-containing protein; *orfB*, HAD family hydrolase; *orfC*, DUF5339 domain-containing protein; *orfD*, GNAT family N-acetyltransferase; *orfE*, DUF3343 domain-containing protein; *orfF*, glyoxalase; *orfG*, carboxymuconolactone decarboxylase family protein; *orfH*, DMT family protein; *orfI*, DMT family transporter; *orfJ*, aminoglycoside 6’-acetyltransferase; *orfK*, SMI1/KNR4 family protein; *orfL*, GNAT family N-acetyltransferase; *orfM*, SDR family NAD(P)-dependent oxidoreductase; *orfN*, MerR family transcriptional regulator; *orfO*, methyl-accepting chemotaxis protein; *orfP*, cytosine permease; *orfQ*, glyoxalase/bleomycin resistance/dioxygenase family protein.

To investigate the distribution of *fosA13*-homologous genes, the nucleotide sequence of *fosA13* was used as a query to search for similar genes in the NCBI nucleotide database. As a result, a total of 82 similar genes with similarities between 87.3% and 100.0% were obtained, all of which were derived from the *M. morganii* genomes (**Supplementary Table S3**). Among these 82 genes, only one had a similarity of 87.3%, and all the others had similarities of ≥ 92.13%. The gene context of the *fosA13*-homologous genes was further analyzed. The 20-kb sequences with the *fosA13*-homologous genes at the center were intercepted and clustered. Finally, these 83 20-kb fragments (including the gene identified in this study) were grouped into 13 clusters with a similarity threshold of 90.0%. The gene context of 13 sequences consisting of one from each of the 13 clusters was compared (**Figure 3**, **Supplementary Table S3**).

As mentioned above and illustrated in **Figure 3**, the *fosA13* and *fosA13*-homologous genes and their surrounding sequences were relatively conserved in the *M. morganii* genomes. Further structural analysis revealed that no mobile genetic element (GME) was present in their flanking regions. Upstream of *fosA13* are genes encoding proteins related to *nifj* [ferredoxin (flavodoxin) oxidoreductase], *fldI* [phenyllactate dehydratase activator] and *fld* [(R)-phenyllactyl-CoA dehydratase beta subunit], whereas downstream of *fosA13* are genes related to *pcpR* (PCP degradation transcriptional activation protein), *yijE_1* (putative cystine transporter YijE_1) and *perR* (HTH-type transcriptional regulator PerR). However, many other *fos* genes, such as *fosC2* (Wachino et al., 2010), *fosA3* (Wachino et al., 2010), *fosA5* (Ma et al., 2015), *fosA6* (Guo et al., 2016) and *fosA8* (Poirel et al., 2019), are related to MGEs and are encoded on plasmids.

## Conclusion

This paper presents the discovery of a novel chromosomal fosfomycin resistance gene, designated *fosA13*, from an animal *M. morganii* isolate. Although *fosA13* encodes a protein that shares less than 80% aa similarity with functionally characterized FosA proteins, the function and structure essential residues of these proteins are conserved within it. Many *fosA*-type genes are located on plasmids of different bacterial species. These genes are easily captured by mobile genetic elements and transmitted between bacteria of different species by means of horizontal gene transfer, which results in widespread resistance. Identifying more resistance mechanisms would greatly benefit the treatment of bacterial infections in the clinic and the monitoring of resistance transmission.

## Data Availability

The datasets presented in this study can be found in online repositories. The names of the repository/repositories and accession number(s) can be found in the article/**Supplementary Material.**
