# Supplementary material for: Characterization of FosA13, a novel fosfomycin glutathione transferase identified in a Morganella morganii isolate from poultry
Source: Front Cell Infect Microbiol. 2025 Mar 11;15:1534084. doi: 10.3389/fcimb.2025.1534084 (PMC11933065; doi:10.3389/fcimb.2025.1534084)
Supplement: Supplementary file 1 [file Table1.docx]

TABLE S1 | The MIC results of the reference strains (μg/mL).

| Bacterium | Source | Original isolate | Recombinant | Fold increase compared to the recepient |
| --- | --- | --- | --- | --- |
| *Salmonella Heidelberg* (*fosA7*) | Chicken | / | ＞512 | 256 |
| *E. coli* (*fosA6*) | Human | 512 | 12 | 32 |
| *Mycobacterium abscessus* (*fosI*) | Human | ＞1024 | 16 | 128 |
| *Staphylococcus aureus* (*fosY*) | Human | 8 | 16 | 16 |
