# Supplementary material for: Characterization of FosA13, a novel fosfomycin glutathione transferase identified in a Morganella morganii isolate from poultry
Source: Front Cell Infect Microbiol. 2025 Mar 11;15:1534084. doi: 10.3389/fcimb.2025.1534084 (PMC11933065; doi:10.3389/fcimb.2025.1534084)
Supplement: Supplementary file 2 [file Table2.docx]

TABLE S2 | Resistance genes identified in the *Morganella morganii* DW0548 genome.

| Drug class | Gene Name | Coverage (%) | Identity (%) |
| --- | --- | --- | --- |
| Chloramphenicol | *catII* | 100.0 | 97.7 |
| beta-lactam | *bla*_DHA-16_ | 100.0 | 99.7 |
