# Supplementary material for: Characterization of FosA13, a novel fosfomycin glutathione transferase identified in a Morganella morganii isolate from poultry
Source: Front Cell Infect Microbiol. 2025 Mar 11;15:1534084. doi: 10.3389/fcimb.2025.1534084 (PMC11933065; doi:10.3389/fcimb.2025.1534084)
Supplement: Supplementary file 3 [file Table3.docx]

TABLE S3 | Clustering result of the 83 *fosA13-*homologous gene carrying sequences.

| Cluster | Source | Similarity (%) | Accession No. |
| --- | --- | --- | --- |
| 1 | *Morganella morganii* DW0548 | 100 | CP173707.1 |
|  | *Morganella morganii strain* FAM24676 | 96.76 | CP066132.1 |
| 2 | *Morganella morganii strain* 11759 | 92.82 | CP059986.1 |
|  | *Morganella morganii strain* DSM 30164 | 92.82 | CP069157.1 |
|  | *Morganella morganii strain* HAMBI_1292 | 92.82 | CP139988.1 |
|  | *Morganella morganii strain* Jiangxi | 92.82 | CP039377.1 |
|  | *Morganella morganii strain* K266 | 92.82 | CP103768.1 |
|  | *Morganella morganii strain* MM1680 | 92.82 | CP149979.1 |
|  | *Morganella morganii strain* MM4205 | 92.82 | CP149978.1 |
|  | *Morganella morganii strain* MM6005 | 92.82 | CP149984.1 |
|  | *Morganella morganii strain* N18-00103 | 92.82 | CP048275.1 |
|  | *Morganella morganii strain* NCTC235 | 92.82 | LR133904.1 |
|  | *Morganella morganii strain* PS005-13 | 92.82 | CP151817.1 |
|  | *Morganella morganii strain* RB11 | 92.59 | CP150714.1 |
|  | *Morganella morganii strain* S164-3 | 92.82 | CP059477.1 |
|  | *Morganella morganii subsp. morganii strain* 229813 | 92.82 | CP043955.1 |
|  | *Morganella morganii subsp. morganii strain* ZJC25 | 92.82 | CP064828.1 |
| 3 | *Morganella morganii strain* 2022CK-00284 | 92.36 | CP132323.1 |
|  | *Morganella morganii strain* 2387 | 92.82 | CP139441.1 |
|  | *Morganella morganii strain* DG56-16 | 92.59 | CP032295.1 |
|  | *Morganella morganii strain* FDAARGOS_172 | 92.82 | CP014026.2 |
|  | *Morganella morganii strain* L241 | 92.82 | CP033056.1 |
|  | *Morganella morganii strain* MM4512 | 92.82 | CP149985.1 |
|  | *Morganella morganii strain* MMAS2018 | 93.06 | CP086203.1 |
|  | *Morganella morganii strain* MP63 | 92.59 | CP048806.1 |
|  | *Morganella morganii strain* NCTC12028 | 92.82 | LS483498.1 |
|  | *Morganella morganii strain* RB03 | 92.82 | CP150716.1 |
|  | *Morganella morganii strain* RB05 | 92.82 | CP150715.1 |
|  | *Morganella morganii strain* RB07 | 92.59 | CP150713.1 |
|  | *Morganella morganii strain* SDTA-1 | 93.06 | CP134699.1 |
|  | *Morganella morganii strain* SMM01 | 92.82 | CP063843.1 |
|  | *Morganella morganii subsp. morganii strain* 12304 | 92.82 | CP064832.1 |
|  | *Morganella morganii subsp. morganii strain* 516602 | 92.82 | CP064054.1 |
|  | *Morganella morganii subsp. morganii strain* ATCC 25830 | 92.82 | CP034944.1 |
|  | *Morganella morganii subsp. morganii* KT | 92.82 | CP004345.1 |
|  | *Morganella morganii subsp. morganii strain* ZJG812 | 92.59 | CP064831.1 |
|  | *Morganella morganii subsp. morganii strain* ZJG944 | 92.59 | CP064827.1 |
|  | *Morganella morganii strain* UM869 | 92.36 | CP104700.1 |
| 4 | *Morganella morganii strain* 2022JQ-00674 | 92.13 | CP137459.1 |
|  | *Morganella morganii strain* AR_0057 | 92.59 | CP027177.1 |
|  | *Morganella morganii strain* Colony180 | 92.13 | CP076370.1 |
|  | *Morganella morganii strain* FDAARGOS_365 | 92.59 | CP023505.1 |
|  | *Morganella morganii strain* FDAARGOS_63 | 92.13 | CP026046.1 |
|  | *Morganella morganii isolate* GN28 | 92.13 | CP026651.1 |
|  | *Morganella morganii strain* KC-Tt-01 | 92.36 | CP025933.1 |
|  | *Morganella morganii strain* MM2467 | 92.13 | CP149991.1 |
|  | *Morganella morganii strain* MM46903 | 92.59 | CP070524.1 |
|  | *Morganella morganii strain* MM48659 | 92.13 | CP070537.1 |
|  | *Morganella morganii strain* MM50821 | 92.59 | CP070553.1 |
|  | *Morganella morganii strain* MM9291 | 92.13 | CP149988.1 |
|  | *Morganella morganii strain* OT11 | 92.59 | CP101057.1 |
|  | *Morganella morganii strain* Sample-M-2023 | 93.29 | CP126137.1 |
|  | *Morganella morganii subsp. morganii strain* 621164 | 92.59 | CP064829.1 |
|  | *Morganella morganii subsp. morganii strain* 715394 | 92.31 | CP064833.1 |
|  | *Morganella morganii subsp. morganii strain* GN28 | 92.13 | CP064055.1 |
|  | *Morganella morganii strain* Z22-3 | 92.13 | CP159742.1 |
| 5 | *Morganella morganii strain* 23 | 92.59 | CP132600.1 |
|  | *Morganella morganii strain* FDAARGOS_1085 | 92.59 | CP068145.1 |
|  | *Morganella morganii subsp. morganii strain* 81703 | 93.06 | CP064830.1 |
|  | *Morganella morganii subsp. morganii strain* QLYYMMQL588 | 92.59 | CP128435.1 |
| 6 | *Morganella morganii strain* 41 | 93.06 | CP132746.1 |
|  | *Morganella morganii strain* RB02 | 93.06 | CP150717.1 |
|  | *Morganella morganii strain* RB04 | 93.06 | CP150712.1 |
|  | *Morganella morganii subsp. morganii strain* ZJD581 | 93.06 | CP064826.1 |
| 7 | *Morganella morganii strain* A19 | 92.36 | CP135144.1 |
|  | *Morganella morganii strain* CTX51T | 95.14 | CP076623.1 |
| 8 | *Morganella morganii strain* DZ1 | 92.36 | CP148043.1 |
| 9 | *Morganella morganii strain* FAM24091 | 100 | CP066777.1 |
|  | *Morganella morganii strain* FAM24206 | 100 | CP066142.1 |
|  | *Morganella morganii strain* FAM24670 | 100 | CP066140.1 |
|  | *Morganella morganii strain* FAM24671 | 100 | CP066138.1 |
|  | *Morganella morganii strain* FAM24672 | 100 | CP066137.1 |
|  | *Morganella morganii strain* FAM24675 | 100 | CP066133.1 |
|  | *Morganella morganii strain* FAM24678 | 100 | CP066130.1 |
|  | *Morganella morganii strain* FAM24681 | 100 | CP068562.1 |
|  | *Morganella morganii strain* FAM24685 | 100 | CP066127.1 |
| 10 | *Morganella morganii strain* FAM24679 | 93.06 | CP066129.1 |
|  | *Morganella morganii strain* HIS2824 | 92.36 | OX460951.1 |
|  | *Morganella morganii subsp. sibonii* SU8481 | 92.36 | AP028645.1 |
| 11 | *Morganella morganii strain* GDMM86 | 93.98 | CP061513.1 |
| 12 | *Morganella morganii strain* Colony326 | 93.29 | CP070407.1 |
|  | *Morganella morganii strain* Colony456 | 92.59 | CP070409.1 |
| 13 | *Morganella morganii strain* AR_0133 | 87.32 | CP028956.1 |
